# Emotional Self-Regulation in Everyday Life: A Systematic Review

**DOI:** 10.3389/fpsyg.2022.884756

**Published:** 2022-05-24

**Authors:** Marina Alarcón-Espinoza, Susana Sanduvete-Chaves, M. Teresa Anguera, Paula Samper García, Salvador Chacón-Moscoso

**Affiliations:** ^1^Departamento de Psicología, Universidad de La Frontera, Temuco, Chile; ^2^Departamento de Psicología Experimental, Universidad de Sevilla, Seville, Spain; ^3^Faculty of Psychology, Institute of Neurosciences, University of Barcelona, Barcelona, Spain; ^4^Department of Basic Psychology, University of Valencia, Valencia, Spain; ^5^Departamento de Psicología, Universidad Autónoma de Chile, Santiago, Chile

**Keywords:** evolutionary development, emotional regulation, observational methodology, natural contexts, childhood, adolescence

## Abstract

Emotional self-regulation in childhood and adolescence constitutes a growing interest in the scientific community, highlighting in recent years the need to observe its development in their daily life. Therefore, the objective of this systematic review is to characterize publications referring to the development of emotional self-regulation of people under 18 years-old, in natural contexts. Based on the PRISMA guidelines, searches are carried out in the Web of Science, Scopus and PsycINFO databases, and in Google Scholar until May 2020. After reviewing the full text of 376 publications, 14 works are selected that are observed in their extrinsic, substantive and methodological characteristics based on the GREOM and MQCOM guidelines, by two independent evaluators. Most of the studies correspond to the last 20 years, increasing the interest in observing older children, in interaction with adults and/or in different cultures. They apply mixed methodologies, not always ascribing to a low intensity design. Strengths are observed regarding the collection and analysis of the quality of the data; and weaknesses related to the failure to record the duration and sequence of behaviors, highlighting the use of guidelines as guides for future research.

## Introduction

Emotional self-regulation, referring to the understanding, acceptance, and modulation of emotional responses, is a process that children and adolescents carry out in order to adapt to their psychosocial environment, orienting themselves toward the achievement of their evolutionary goals and favoring their mental health (Van Lissa et al., 2019). The achievement of emotional self-regulation allows progress in the acquisition of greater autonomy, at the same time that it is related to the development of adequate self-esteem and feelings of self-efficacy that facilitate social and school adjustment. The emotional educational process is continuous and permanent throughout the life cycle (Gallardo Fernández and Saiz Fernández, 2016), favoring the individual in order to achieve emotional competence which allows to regulate their emotions.

Much of the research on emotional development has focused on the relationships between parents and children under the age of two and/or preschoolers. However, in the last decade there have been studies referring to understanding how families socialize the expression of emotions in their children’s middle childhood and adolescence (Adrian et al., 2011; Bai et al., 2016), observing that the emotional regulation level that children reach at age 7 predicts the quality of positive friendship at age 10, showing greater ability to express their emotions effectively, interpret emotions and respond to them appropriately (Blair et al., 2014).

Gallardo Fernández and Saiz Fernández (2016) have highlighted that the 21st century school has to assume responsibility for educating children’s emotions as much or more than the family, highlighting that educators must be the main emotional leaders of the students. Furthermore, Bailey et al. (2016) highlight that children marked by effective interactions with their teachers have better socio-emotional and cognitive skills, highlighting that effective teachers can help children in the transition toward self-regulation of their emotions; and that the emotional and organizational support of the educational context can be particularly sensitive to the social-emotional functioning of children in the classroom.

Meanwhile, in the field of research, Sabatier et al. (2017) point out that, in the last 15 years of research in the field of emotional development, the findings regarding neurobiological and environmental elements that influence the acquisition of skills to manage emotions have been highlighted, with consensus that, with age, people improve in the control of their emotions. However, fewer studies were observed that analyze these regulatory processes during the adolescent period and many of these studies would correspond to western and developed countries. Also, there is a need to document the development of emotional regulation processes in different social and economic contexts.

Compas et al. (2017), based on a meta-analytic review, propose an agenda for future research that includes improving the conceptualization seeking integration between the various constructs that study the subject; prioritize the study of the development of emotional regulation capacities instead of the study of symptoms, and improve the methodology and research designs by approaching more ecological models that allow understanding these processes in real contexts and times.

Likewise, Adrian et al. (2011) affirm that the empirical evidence indicates that emotional regulation skills are developed in a dynamic and multifaceted system, observing that, although observational and longitudinal methodologies have been mostly used with children under 6 years of age, it is necessary to continue carrying out multimodal evaluations and research with multiple methods and multilevel assessments in school-age children and adolescents.

Along the same lines, Buckley et al. (2003), emphasize the need to research about the emotional development of children and adolescents, in a collaborative way with school personnel, thus being able to observe how they use different coping strategies in natural development contexts, an aspect that is also mentioned by Bai et al. (2016), who have also emphasized the need to know how children’s spontaneous emotional expressions develop and maintain in uncontrolled environments of daily life, particularly within the family and during the school-age years.

Therefore, understanding natural contexts as all those contexts in which the behavior is habitual, and is not constrained by requirements that alter spontaneity (Craik, 2000; Bolger et al., 2003; Wilhelm et al., 2012), the objective of this systematic review has been to characterize publications referring to the development of emotional self-regulation in people under the age of 18 years, through relationship/communication guidelines, in natural contexts.

## Methods

Bibliographic searches were carried out in the Web of Science, Scopus and PsycINFO databases and in academic Google from its inception until May 2020 with the following keywords in title, keywords and/or abstract: (“emotional autoregulation”) OR (“autorregulación emocional”) OR (“emotional self-regulation”) OR (“emotional selfregulation”) OR (“emotional self regulation”) OR (“competencias emocionales”) OR (“emotional skills”) OR (“emotional competences”) OR (“regulación emocional”) OR (“emotional regulation”) OR (“educación emocional”) OR (“emotional education”) AND (“comunicación”) OR (“communication”) AND (“relaciones interpersonales”) OR (“relationships”) AND (“vida cotidiana”) OR (“daily life”).

As inclusion criteria of the studies selected to respond to the objective of the present investigation, the following were considered: (a) that their objective was to investigate self-regulation/emotional regulation; (b) primary studies, excluding theoretical works, systematic reviews, and meta-analysis; (c) that they observe daily relationship/communication patterns in natural contexts; (d) that the main participants were people under 18 years of age (regardless of whether parents or teachers were also involved) (e) who studied universal population (normal evolutionary development); (f) written in English or Spanish; (g) with access to the full text. For study selection, two investigators applied the criteria independently. Subsequently, intercoder reliability was calculated using the kappa coefficient (κ). Agreement was reached on the discrepancies found with the mediation of a third researcher.

Additionally, in order to expand the number of primary studies included, the references of the included texts were reviewed and the authors were written to in order to request new articles that could meet the criteria indicated.

The included works were reviewed in order to observe: (1) extrinsic characteristics: their institutional affiliation, type and year of publication, and country where the research was carried out; (2) substantive characteristics: referring to the characteristics of the sample, and the way to conceptualize, base and evaluate self-regulation/emotional regulation; and (3) methodological characteristics: recording the characteristics of the method explicitly declared by the authors; those observed according to *Guidelines Reporting Evaluations based on Observational Methodology GREOM* (Portell et al., 2015); and those observed according to *Methodological Quality Checklist for studies based on Observational Methodology (MQCOM*; Chacón-Moscoso et al., 2019).

The review of the articles was carried out independently by two researchers, who when applying the MQCOM guide, had to agree on their observations in the face of the discrepancies found with the arbitration of a third expert researcher. The degree of initial agreement was calculated with the coefficient κ.

## Results

### Selection of Studies

**FIGURE 1:**
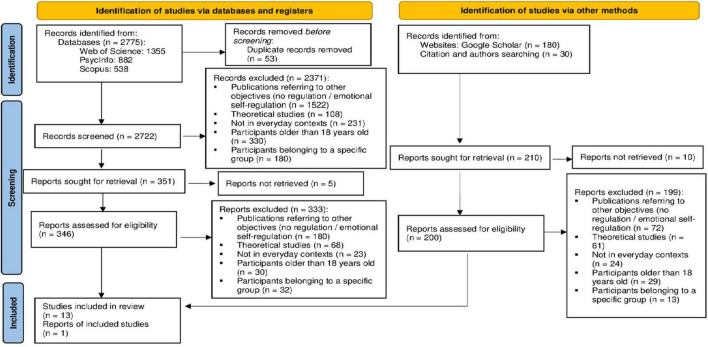
Study selection flow chart.

Figure 1 presents the PRISMA flow chart (Page et al., 2021), with the selection process of the primary studies in the systematic review. The intercoder reliability in the selection obtained a κ = 0.73. Finally, 14 studies met the inclusion criteria.

### Characteristics of the Studies

In the coded variables, the intercoder reliability reached κ = 0.81.

Regarding the extrinsic characteristics of the articles studied (see Table 1), there is a progressive increase in publications with the mentioned inclusion criteria, the first of which was observed in 1999. The institutional affiliation of the researchers corresponds mainly to North American universities (8), observing two works by George Mason University. Of the other works, two publications report the joint effort of researchers from different universities, both of whom are the same researcher from the University of Osnabrück in Germany.

**TABLE 1:** Extrinsic characteristics.

References	Institutional affiliation	Publication type	Country of the experience
Bai et al. (2016)	University of California, Los Angeles; McLean Hospital, Belmont, Massachusetts and Harvard Medical School	Article	United States
Bailey et al. (2016)	Yale University; George Mason University	Article	United States
Fabes et al. (1999)	Department of Family Resources & Human Development, Arizona State University; Arizona State University	Article	United States
Feldman and Klein (2003)	Department of Psychology, Bar-Ilan University, Israel	Article	Israel
Gallegos et al. (2017)	University of Texas at Austin	Article	United States
Garner (2006)	New Century College, George Mason University, 4400 University Drive	Article	United States
Herndon et al. (2013)	George Mason University	Article	United States
Keller et al. (2004)	University of Osnabrück, Germany; Henning Jensen, University of Costa Rica, San Jose; Aristotle University of Thessaloniki, Greece	Article	Cameroon, Greece and Costa Rica
Kim et al. (2014)	The Pennsylvania State University	Article	United States
Kochanska et al. (2007)	University of Iowa	Article	United States
Lavelli et al. (2019)	University of Verona; University of Milano-Bicocca; University of Osnabrück, Germany and Hebrew University of Jerusalem	Article	Italy, Nigeria, Ghana and Cameroon
Roberts (2020)	York University, Toronto, Ontario, Canada	Article	Canada
Santa Cruz (2020)	Universidad Cesar Vallejo, Trujillo, Peru	Bachelor thesis	Peru
Silva et al. (2018)	Universidade do Minho, Portugal	Article	Portugal

Regarding the substantive characteristics of the reviewed papers (see Table 2), most of the articles look at preschool-age children. Regarding the ethnicity variable, it is observed that 8 of the studies make explicit mention of the race of the participants and that none of them refer to the comparison between different cultures or countries. Studies interested in observing cultural variables indicate the nationality and/or immigration status of the participants.

**TABLE 2:** Substantive characteristics.

Publication	Sample size			Age	Gender	Race or Nationality
	Under 18 years	Fathers and/or mothers	Teachers			
Bai et al. (2016)	31	29 mothers and 31 fathers	–	8–12 years Fathers M:41,5 (SD:5,6)	14 girls and 17 boys	European Americans (64.5%), Mestizos (19.4%), Asian Americans (9.7%), Latino (3.2%), African American (3.2%)
Bailey et al. (2016)	312	–	44	3–5 years old	155 boys and 157 girls	52% White, 31% African American, and 2% Asian, Native American, or Pacific Islander; 10% of parents did not report ethnicity
Fabes et al. (1999)	135	–	–	4–5 years old	77 boys, 58 girls	87% Caucasian; 7% Mexican American, 4% African American, 2% Asian
Feldman and Klein (2003)	90	90 mothers and 42 fathers	16	2 years	52 boys and 38 girls	Israelis
Gallegos et al. (2017)	125	125 mothers and 125 fathers	–	Last trimester of pregnancy, 8 and 24 months of children.	74 boys and 51 girls	84% White, 8% Hispanic, 2% African American, 6% biracial or other ethnicity
Garner (2006)	70	70 mothers	–	3–6 years old	52% male	African American
Herndon et al. (2013)	308	–	–	Children 3–5 years old	51.0% male children	57% Caucasian, with 33.6% from African American families. 15% Latino/Hispanic, 6.4% not reporting
Keller et al. (2004)	116	116 families	–	3–20 months	–	Cameroon, Greece and Costa Rica
Kim et al. (2014)	132	132 mothers	–	12 and 18 months	78 (54.2%) women and 54 (37.5%) men	86.1% White, 3.5% African American, 2.1% Asian, 4.9% Latino, and 2.8% Other
Kochanska et al. (2007)	112	112 fathers	–	9 months to 6 years	52 girls	97% White
Lavelli et al. (2019)	60	60 mothers	–	Babies and mothers	Italian: 50% girls; immigrants: 50% girls; Cameroon: 60% girls.	20 Italian mother and child dyads, 20 first-generation West African immigrant mothers and their Italian-born babies, and 20 Cameroonian dyads
Roberts (2020)	33	10 mothers and 26 fathers	–	Families with children from 4 to 6 years old	16 girls and 17 boys	85% Canadian English
Santa Cruz (2020)	73	–	–	3 years old	–	–
Silva et al. (2018)	33	33 families	–	12–18 years old	21 girls (63%)	97% Portuguese, 3% Brazilian

Regarding the objectives of interest of the reviewed works (see Table 3), the reference frameworks used to support the study of emotional regulation come from the last years of the 20th century. The authors, when proposing the objectives of their research, indicate more than one motivation, highlighting the interest in research regarding evolutionary development and interaction/communication within the family.

**TABLE 3:** Specific objectives of interest.

References	Authors cited when defining Emotional Self-Regulation	Objective associated with observing self-regulation or emotional regulation									Self-regulation/emotional regulation assessment					
		Evolutionary development	Academic performance	Discipline or school adjustment	Family interaction/communication	Ethical-moral/civic development	Teaching methodologies	Cultural differences	Social development/prosociality	Prevention mental health difficulties	Instrument with adequate psychometric characteristics	Consider evaluator training	Design tasks to evaluate	Use various evaluation methods	Collect information through Different contexts or informants	Analyze information through inter-judge agreement
Bai et al. (2016)	–Gross				X							X				X
Bailey et al. (2016)	–Pianta –Thompson			X								X	X	X	X	X
Fabes et al. (1999)	–Eisenberg and Fabes –Cummings and Cummings	X							X					X	X	X
Feldman and Klein (2003)	–Vygotsky, Feldman, Greenbaum and Yirmiya	X			X							X	X	X	X	X
Gallegos et al. (2017)	–Morris, Silk, Steinberg, Myers and Robinson	X			X							X	X	X	X	X
Garner (2006)	–Thompson	X			X	X		X	X			X			X	X
Herndon et al. (2013)	–Cole, Michel and Teti –Denham, Zinsser and Brown	X		X							X	X		X		
Keller et al. (2004)	–Kopp	X						X				X	X	X	X	X
Kim et al. (2014)	–Calkins and Leerkes –Kopp –Thompson	X			X					X			X	X	X	X
Kochanska et al. (2007)	–Kopp –Caspi and Shiner	X			X						X	X	X	X	X	X
Lavelli et al. (2019)	–Fogel –Camras, Shuster and Fraumeni	X			X										X	X
Roberts (2020)	–Kopp –Thompson	X			X				X			X		X	X	X
Santa Cruz (2020)	–Goleman –Perpiñán	X		X					X		X					X
Silva et al. (2018)	–Gross –Gilbert	X										X		X	X	

Regarding the observed methodological characteristics (Table 4), in three of the publications the authors describe their work as observational – naturalistic, five studies claim to be longitudinal and six are defined as descriptive. Four of the studies propose observation in the natural context as the only form of evaluation; while the remaining investigations indicate this modality among other possibilities, such as the completion of questionnaires or tasks designed to provoke certain emotions or behaviors. The instruments reported to observe self-regulation/emotional regulation are mostly *ad hoc* observation instruments. The dimensions that the authors are interested in observing refer mainly to the interaction between children and adults.

**TABLE 4:** Methodological characteristics declared by the authors.

References	Design	Data collection technique or instrument	Observed dimensions
Bai et al. (2016)	Naturalistic observational	Video recordings taken in homes and community settings	Mutual display of positive emotion, touch and joint leisure
Bailey et al. (2016)	Descriptive	Classroom observations and teacher questionnaires: Preschool Learning Behaviors Scale (PLBS); Social Competence and Bahavior Evaluation (SCBE-30); Teaching Rating Scale of School Adjustment (TRSSA); Student - Teacher Relationship Scale (STRS) The Preschool Self-Regulation Assessment (PSRA); PSRA-AR; CLASS	School Adjustment, Executive Control, Emotional Regulation, Emotional and Organizational Support
Fabes et al. (1999)	Descriptive	Observations of infantile behaviors. Teacher questionnaires (CBQ). Social competence teacher qualifications: Scale of Perceived Social Competence for Children.	Intensity of peer interaction Negative Emotions Positive or constructive social interactions
Feldman and Klein (2003)	Observational naturalistic and Descriptive	Observation of the relational style of the adult: Coding Interactive Behavior (CIB). Observation of Mediational Interaction (OMI). Interviews with caregivers. Observation of child compliance and adult discipline: The Observer. Self-regulation observation of childhood emotion (cognitive tests). Quality observation of the caregiver’s relational style in the group.	Child Compliance Self-regulatory compliance of mothers and caregivers Childhood cognition and the regulation of emotions
Gallegos et al. (2017)	Longitudinal	Video recordings of couple’s discussion tasks. Observations of video interactions in games and routines: child care scales (ICS), classification method by criteria, emotional abstinence scale. Observation of co-parenting conflict (CFRS), verbal sparring scale (ICC = 0.74), a measure of co-parenting conflict Observation of the emotional regulation of children	Negative marital affect observed before birth Parental emotional withdrawal Coparenting conflict Child regulation
Garner (2006)	Naturalistic observational	Observations in home visits Observations in visits to preschool centers	Prosocial socialization behaviors Socialization behaviors of emotions Peer episodes that caused emotion dysregulation
Herndon et al. (2013)	Descriptive	Observation of socio-emotional behavior: Minnesota Preschool Affection Checklist (MPAC-R/S). Teacher Qualifications: SCBE-30; PLBS; STRS; TRSSA	Child socio-emotional behaviors Attitudes toward school Positive relationships among teachers Cooperative participation
Keller et al. (2004)	Longitudinal	Observation of breeding systems Self recognition: blush test. Observation of self-regulation: Fulfillment of requests and Fulfillment of the prohibition	Breeding systems Auto-recognition Self-regulation
Kim et al. (2014)	Longitudinal	Observation of emotional regulation Video observation at bedtime: Emotional availability scales (EAS). Child attachment security: Strange situation. Childish temperament: Revised Infant Behavior Questionnaire (IBQ-R) and Early Infant Behavior Questionnaire (ECBQ)	Mothers’ emotional availability Child attachment security Emotional regulation strategies
Kochanska et al. (2007)	Longitudinal	Observation of mother-child dyad Positive Emotionality Laboratory Procedures for Children Children’s Intellectual Functioning: WPPSI-R information scale Children’s impulsiveness: CBQ	Positive emotionality Self-regulation Intellectual functioning of the child
Lavelli et al. (2019)	Longitudinal	Observation of emotional expression in social interaction. Observation of maternal behaviors.	Active child care Maternal gaze and facial behaviors
Roberts (2020)	Descriptive	Parenting Practices Q-sort Parenting Questionnaire Observation of interactions between peers and friendship networks. Questionnaire for teachers and observers Q-Sort. Observation at home: Individual focal samples	Episodes of childhood distress Parents’ approach to distress Children’s social competence
Santa Cruz (2020)	Descriptive	Checklist of children’s behaviors in free play and emotional self-regulation	Self-regulation of emotions in students
Silva et al. (2018)	Descriptive	Daily life reports. Perceptions questionnaire on sampling week Emotional regulation questionnaire: ERQ-CA. Positive and negative affect status questionnaire Self-observation of emotional regulation strategies	Emotional regulation strategies

Regarding the methodological characteristics observed by GREOM –first part– (Table 5), five publications justify the choice of a low intensity observation method. Regarding the study units, four publications indicate and apply inclusion criteria. Regarding the observed sessions, eight articles indicate the period of time in which it has been observed, seven specify the number of observation sessions carried out, seven publications mention the period of time elapsed between the observations, and eleven inform the method used for sampling. All publications describe the observation instrument used, six justify it, ten provide access to the instrument and one provides access to the coding manual.

**TABLE 5:** Methodological characteristics observed through the Guidelines for Reporting Evaluations based on Observational Methodology -GREOM- (first part).

References	Observation method justification	Description of expected results	Design description			Inclusion criteria indicated and applied	Times		Contexts		Observation instrument								

			Informs observational design	Justify observational design	Participants you want to observe	Sequential data	Observation of common contexts	Specify the observation period	Specify number of observation sessions	Specifies the periodicity between observation sessions	Specify method used for sampling	Indicate WHAT is observed	Indicate WHO is being observed	Indicate CIRCUMSTANCES observed	Describe the observation instrument	Justify the observation instrument used	Provide access to the observation instrument used	Provide access to the encoding manual used	
Bai et al. (2016)	X	X		X	Several	X	X	X	X	X	X	X	X	X	X	X	X	X	
Bailey et al. (2016)		X		Partial	Several	X	X		X	X			X	X	X	X		X	
Fabes et al. (1999)	X	X		Partial	Several	X	X		X	X	X	X	X	X	X	X			
Feldman and Klein (2003)		X		Partial	Several		X					X	X	X	X	X			
Gallegos et al. (2017)		X		Partial	Several		X						X	X	X	X		X	
Garner (2006)		X		Partial	Several	X	X	X	X		X	X	X	X	X	X			
Herndon et al. (2013)		X		Partial	Several		X		X	X	X	X	X	X	X	X	X	X	
Keller et al. (2004)		X		Partial	Several		X	X	X	X		X	X	X	X	X	X	X	
Kim et al. (2014)	X	X		Partial	Several		X					X	X	X	X	X		X	
Kochanska et al. (2007)		X		Partial	Several		X			X	X	X	X	X	X	X			
Lavelli et al. (2019)		X		Partial	Several	X	X	X	X		X	X	X	X	X	X	X	X	X
Roberts (2020)	X	X		Partial	Several		X					X	X	X	X	X		X	
Santa Cruz (2020)		X		Partial	Several		X						X	X	X	X	X	X	
Silva et al. (2018)	X	X		Partial	Several	X	X		X	X	X	X	X	X	X	X	X	X	

*It is marked with an X when the criterion is met.*

Regarding the primary recording parameters, all of them record frequency (GREOM second part, see Table 6), five record the duration of the behavior and three mention the behavioral sequences. The information is recorded mainly through videos and observed by trained personnel. In relation to data quality control, thirteen studies report concordance analysis of the collected data. Regarding the analysis of the data carried out, all of them made explicit the type of analysis used and thirteen of them justified it.

**TABLE 6:** Methodological characteristics observed through Guidelines for Reporting Evaluations based on Observational Methodology -GREOM- (second part).

References	Primary recording parameters			Means of observation	Session acceptance criteria		Observer characteristics					Reliability	Flow of study units		Analysis	
	Frequency	Duration	Sequence		Justification of consistency between sessions	Justification of interruptions of the sessions	The observer is a close person	The observer has been trained	The observer is being evaluated	The observer receives a payment	Self-report		Report observation interruptions	Report withdrawals from participants	Data analysis used	Justify data analysis modality
Bai et al. (2016)	X	X		Video	X	X		X				X	X	X	X	X
Bailey et al. (2016)	X			Video				X				X			X	X
Fabes et al. (1999)	X			Video	X			X				X	X		X	X
Feldman and Klein (2003)	X	X		Video				X				X			X	X
Gallegos et al. (2017)	X			Video				X				X		X	X	X
Garner (2006)	X		X	Audio and pencil and paper				X				X		X	X	X
Herndon et al. (2013)	X			Pencil and paper				X				X			X	X
Keller et al. (2004)	X			Video				X				X	X	X	X	X
Kim et al. (2014)	X			Video				X				X		X	X	
Kochanska et al. (2007)	X	X		Video				X				X		X	X	X
Lavelli et al. (2019)	X	X	X	Video	X		X	X				X			X	X
Roberts (2020)	X	X		Video				X				X			X	X
Santa Cruz (2020)	X			Pencil and paper								X			X	X
Silva et al. (2018)	X		X	Mobile							X				X	X

*It is marked with an X when the criterion is met.*

Regarding the methodological characteristics observed through MQCOM (see Table 7), seven studies justify and support the observation methodology used based on the degree of perceptiveness of the information. In one of the investigations, software is used to record, control, and analyze the quality of the data, and in four investigations, the use of this tool was partial. Regarding the type of parameters recorded, in 10 studies the secondary record derived from the recording of a single category (for example: frequency or duration) was observed, in two studies the primary record of a single category was observed, and in the other two investigations the dynamic or transition recording between different observation parameters was used.

**TABLE 7:** Methodological characteristics observed using the Methodological Quality Checklist for studies based on Observational Methodology (MQCOM).

References	Reference to the observation methodology	Delimitation of the study objectives	Referenced theoretical framework	Observation unit criteria	Temporal criteria	Dimensionality criteria	Inclusion/exclusion criteria	Adequacy of the observation instrument	Coding manual	Software usage	Data type specification	Parameters specification	Session delimitation	Inter-observer reliability	Type of data analysis	Interpretation of results in the discussion
Bai et al. (2016)	0.5	1	1	1	1	1	1	1	1	0.5	–	0.5	1	1	1	1
Bailey et al. (2016)	1	0.5	1	1	0.5	1	1	1	1	0.5	–	0.5	0.5	1	1	1
Fabes et al. (1999)	0.5	1	1	1	0.5	1	0.5	1	0.5	0	–	0.5	1	1	1	1
Feldman and Klein (2003)	0.5	1	1	1	0.5	1	0.5	1	1	0	–	0.5	1	1	1	1
Gallegos et al. (2017)	0.5	1	1	1	1	1	1	1	0	0	–	0.5	1	1	1	1
Garner (2006)	0	1	1	1	0.5	1	1	1	1	0	1	0.5	0.5	1	1	1
Herndon et al. (2013)	0.5	0.5	1	0.5	0.5	1	0.5	1	1	0	–	0	0.5	1	1	1
Keller et al. (2004)	1	1	1	1	1	1	1	1	1	0	–	0.5	1	1	1	1
Kim et al. (2014)	0.5	1	1	1	1	1	1	1	0.5	0.5	–	0.5	1	1	1	1
Kochanska et al. (2007)	1	1	1	1	1	1	1	1	0.5	0	–	0.5	1	1	1	1
Lavelli et al. (2019)	1	1	1	1	0.5	1	1	1	1	1	1	1	0.5	1	1	1
Roberts (2020)	1	1	1	1	0.5	1	0.5	1	1	0.5	–	0.5	0.5	1	1	1
Santa Cruz (2020)	1	1	1	1	0.5	1	1	1	0	0	–	0	0.5	1	1	0.5
Silva et al. (2018)	1	1	1	0.5	0.5	1	1	1	0	0	0.5	1	1	0	1	1

*1 = meets the criteria; 0.5 = partially complies; 0 = does not comply.*

All the investigations indicate having carried out some inferential analysis to analyze the data. In 13 publications, the results are interpreted based on the objectives of the study and the scientific literature, while in the other study the results are interpreted based solely on the objectives of the study.

## Discussion

In the last 30 years, there has been a growing interest in the study of emotional self-regulation in older children and in different contexts and cultures, as suggested by Adrian et al. (2011), Bai et al. (2016), and Chervonsky and Hunt (2019). Regarding the substantive characteristics of the works, it is observed that a large part of the studies consider adults linked to children or adolescents as participants, showing a greater interest in observing the interaction in emotional regulation processes.

The countries and universities that lead the research carried out, as pointed out by Sabatier et al. (2017), correspond mostly to territories with higher income and quality of life, many of which have some tradition in studies of the evolutionary development of children. The influence of North American authors such as Claire Koop, Ross Thompson, James Gross, Susan Calkins, Pamela Cole or Nancy Eisenberg is observed, which could be related to having considered only works written in English and Spanish, suggesting that future studies incorporate written works in other languages.

Regarding the methodological characteristics, as strengths it was observed that most of the studies use different techniques or instruments for data collection; that the instruments designed *ad hoc* have a theoretical basis, are applied by properly trained personnel and have data quality control. In all the primary documents, situations typical of daily life are studied and analyzed, observing in all of them the use of the observational methodology, although there are some variants and diverse denominations, for which it is estimated as a weakness, that more than half of the studies do not propose the choice of a low intensity methodological design, with which they do not necessarily consider the richness involved in observing behaviors of daily life and detailing observation parameters such as duration and sequence of behaviors, aspects that are deemed necessary to observe in future research. In this sense, considering that the observational methodology constitutes a contribution to studies referring to evolutionary development in daily life, there is a need to highlight, in the preparation of future research, the review of the guidelines proposed by Chacón-Moscoso et al. (2019) and Portell et al. (2015), in order to guarantee the methodological quality.

Finally, observing that the study of behaviors in daily life has been gaining space and value when questioning the impact of studies carried out in laboratories (Compas et al., 2017), it is observed that, although every day there are older and better technological instruments that allow observing daily life and with people who are willing to comment on their experiences, it is necessary to regulate the ethical scope of the use of social networks in research, since they could affect the private and public life of the participants.

## Data Availability Statement

The original contributions presented in the study are included in the article/supplementary material, further inquiries can be directed to the corresponding author.

## Author Contributions

MA-E and MTA: idea. MA-E and SS-C: literature review (state of the art). MA-E, SS-C, and SC-M: methodology, data analysis, and results. MA-E, MTA, PS, SS-C, and SC-M: discussion and conclusions. MA-E, PS, and MTA: newsroom (original draft). MTA, SS-C, and SC-M: final revisions. MTA and SC-M: project design and sponsorships. All authors contributed to the article and approved the submitted version.

## Conflict of Interest

The authors declare that the research was conducted in the absence of any commercial or financial relationships that could be construed as a potential conflict of interest.

## Publisher’s Note

All claims expressed in this article are solely those of the authors and do not necessarily represent those of their affiliated organizations, or those of the publisher, the editors and the reviewers. Any product that may be evaluated in this article, or claim that may be made by its manufacturer, is not guaranteed or endorsed by the publisher.
